# Reduced Activity of SRY and its Target Enhancer *Sox9*-TESCO in a Mouse Species with X*Y Sex Reversal

**DOI:** 10.1038/srep41378

**Published:** 2017-02-03

**Authors:** Liang Zhao, Alexander Quinn, Ee Ting Ng, Frederic Veyrunes, Peter Koopman

**Affiliations:** 1 Institute for Molecular Bioscience, The University of Queensland, Brisbane, QLD 4072, Australia; 2 Institut des Sciences de l’Evolution de Montpellier, Université Montpellier II, CNRS, Montpellier, France

## Abstract

In most eutherian mammals, sex determination is governed by the Y-linked gene *Sry,* but in African pygmy mice *Mus minutoides, Sry* action is overridden by a variant X chromosome (X*), yielding X*Y females. We hypothesized that X*Y sex reversal may be underpinned not only by neomorphic X chromosome functionality, but also by a compromised *Sry* pathway. Here, we show that neither *M. minutoides* SRY nor its target, the *Sox9-*TESCO enhancer, had appreciable transcriptional activity in *in vitro* assays, correlating with sequence degradation compared to *Mus musculus* counterparts. However, *M. minutoides* SRY activated its cognate TESCO to a moderate degree, and can clearly engage the male pathway in *M. minutoides* in the wild, indicating that SRY and TESCO may have co-evolved in *M. minutoides* to retain function above a threshold level. We suggest that weakening of the SRY/TESCO nexus may have facilitated the rise and spread of a variant X* chromosome carrying female-inducing modifier gene(s).

In most eutherian mammals with an XX/XY chromosomal system, sex development hinges on the presence or absence of the Y-linked testis-determining gene *Sry*^1^,^2^,^3^. SRY protein is a transcription factor characterized by a 79-amino acid DNA binding domain known as the high mobility group (HMG) domain^4^. When expressed in fetal gonads, SRY protein, together with its partner SF1 (also known as NR5A1), bind to the testis-specific enhancer core element (TESCO) of the target effector gene *Sox9* and upregulate its expression^5^. SOX9 protein in turn initiates a genetic cascade directing the bipotential somatic precursor cells to develop into Sertoli cells^6^, which orchestrate the development of a testis^7^. In the absence of *Sry* expression or upregulation of *Sox9*, the fetal gonads develop as ovaries.

While SRY proteins from different species show strong conservation in the HMG domain, sequences outside the HMG domain are poorly conserved^8^,^9^. Mouse and rat SRY proteins have C-termini that are particularly unusual in that they comprise a bridge domain and a polyglutamine (polyQ) tract encoded by a CAG trinucleotide-repeat microsatellite. We have demonstrated previously that the polyQ tract plays essential roles in male sex determination in laboratory mice (*Mus musculus*) by stabilizing SRY protein and transcriptionally inducing *Sox9* expression *via* activating TESCO^10^,^11^.

An atypical sex determination system has been described in the African pygmy mouse *Mus minutoides*. In this species, regular XX females and XY males exist, but in addition, individuals bearing a normal Y and a variant X (X*) develop as females, despite the presence of the Y chromosome and *Sry*^12^,^13^. While the genetic variation that allows the X* to override the male sex-determining programme has not been identified, the other side of the coin is the question of whether the male sex-determining pathway has been weakened in this species, perhaps rendering it vulnerable to be overridden by X*. In *M. minutoides, Sry* is expressed in embryonic and adult X*Y ovaries at levels higher than in XY testes^13^ (and our unpublished data), suggesting that female development in X*Y animals is unlikely due to the lack of *Sry* expression. Previous analyses of *Sry* in *M. minutoides* have identified no disruptive mutations in the HMG box and part of the bridge domain, and no differences in partial *Sry* sequence between XY males and X*Y females^12^,^14^. However, gross defects in *Sry* are unlikely, given that it must retain its male sex-determining function in XY males, leading us to hypothesize that molecular function of the *Sry* pathway might be more subtly compromised in this species.

In the current study, we test this hypothesis by examining in detail the structure and function of SRY in *M. minutoides*. We find that both SRY and its target enhancer, *Sox9*-TESCO, are strongly debilitated in *M. minutoides,* but they appear to have co-evolved such that they remain able to function together in XY males. We propose a model in which weakening of the SRY/TESCO nexus may have facilitated the rise and stabilization of an X*-based feminizing mechanism in *M. minutoides.*

## Results

### A degraded C-terminal polyQ tract in *M. minutoides* SRY

**Figure 1:**
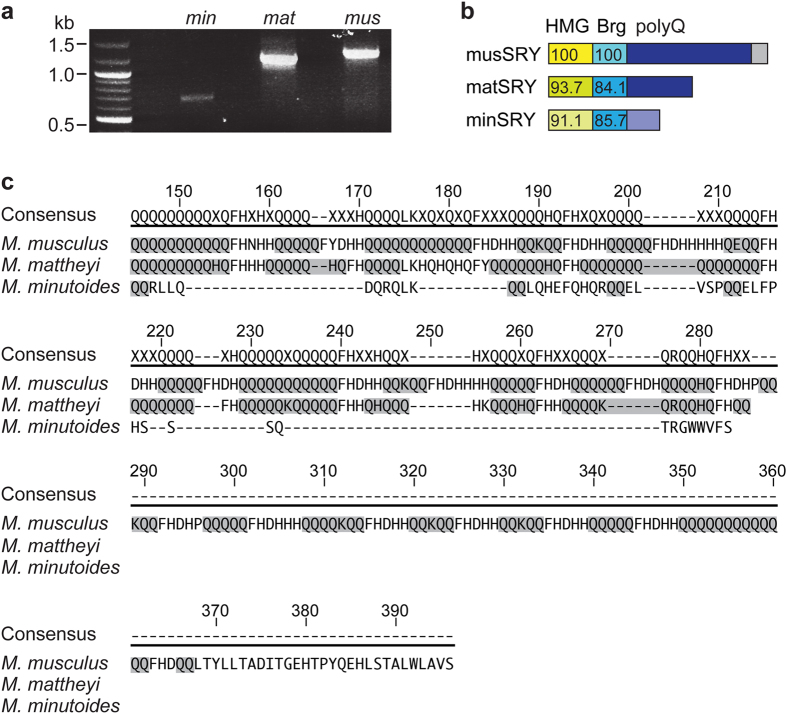
*M. minutoides* SRY has a degraded C-terminal polyQ tract. (**a**) PCR amplification of the full-length *Sry* coding region from *M. minutoides* or *M. mattheyi*. An *M. musculus* sample was included as a control. (**b**) Schematic of SRY proteins from three *Mus* species: *M. musculus* (musSRY), *M. mattheyi* (matSRY), and *M. minutoides* (minSRY). Brg, bridge domain. Numbers indicate the sequence identity scores compared to the same domain of musSRY. (**c**) Alignment of the deduced amino acid sequences of the C-terminal polyQ tract of SRY from the three *Mus* species. Glutamine blocks are highlighted in grey.

We began by investigating whether structural changes in SRY might contribute to the mechanism of X*Y sex reversal. We sequenced the entire *Sry* coding region in *M. minutoides* and *M. mattheyi* (Fig. 1a), a close relative with a typical XX/XY system^15^, and identified 5 and 7 *Sry* haplotypes from *M. minutoides* and *M. mattheyi* respectively (Supplementary Figs S1–4 and Table 1), consistent with previous reports of multiple non-identical *Sry* copies in these species^14^,^16^. In subsequent analyses, a reference clone representing the respective consensus of all identified *Sry* haplotypes in *M. mattheyi* or *M. minutoides* was used (Table 1).

Mouse SRY protein comprises an N-terminal HMG domain responsible for DNA binding, a short bridge domain of unknown function, and a large C-terminal polyQ domain composed of 8 (*Mus musculus domesticus*; hereafter referred to as *M. domesticus*) to 20 (*Mus musculus molossinus*; hereafter referred to as *M. musculus*) blocks of 2–13 glutamine residues interspersed by a short histidine-rich spacer sequence^17^,^18^. Compared with *M. musculus* SRY (musSRY), most sequence variations in *M. mattheyi* and *M. minutoides* SRY (mat- and minSRY) were found in the C-terminal polyQ tract, whereas the HMG box and bridge domains are relatively conserved (Fig. 1b,c, and Supplementary Figs S5,S6), consistent with previous analyses^12^,^14^. The C-terminus of matSRY, despite the truncation caused by an internal stop codon (Supplementary Fig. S5), retains 11 glutamine blocks and histidine-rich spacers, resembling a typical SRY polyQ tract (Fig. 1c). In contrast, the C-terminus of minSRY was further shortened and contains 4 blocks of only 2 glutamine residues and no histidine-rich spacers (Fig. 1b,c).

The fact that other murine species, like *Rattus*, Arvicanthini tribe, and other *Mus* species including pygmy mice share a long glutamine-rich C-terminal domain^18^,^19^ (and our unpublished data) suggests the *M. minutoides* polyQ tract has evolved by degradation of a longer and more organized polyQ tract present in common ancestors to *M. minutoides, M. mattheyi* and *M. musculus*. Supporting this view, further degradation of the polyQ tract has occurred in some of the identified *Sry* haplotypes when compared with the reference ones (*M. minutoides* haplotype *a* and *M. mattheyi* haplotypes *c, g*; Table 1 and Supplementary Figs S1–4).

### Loss of transactivation ability of *M. minutoides* SRY

**Figure 2:**
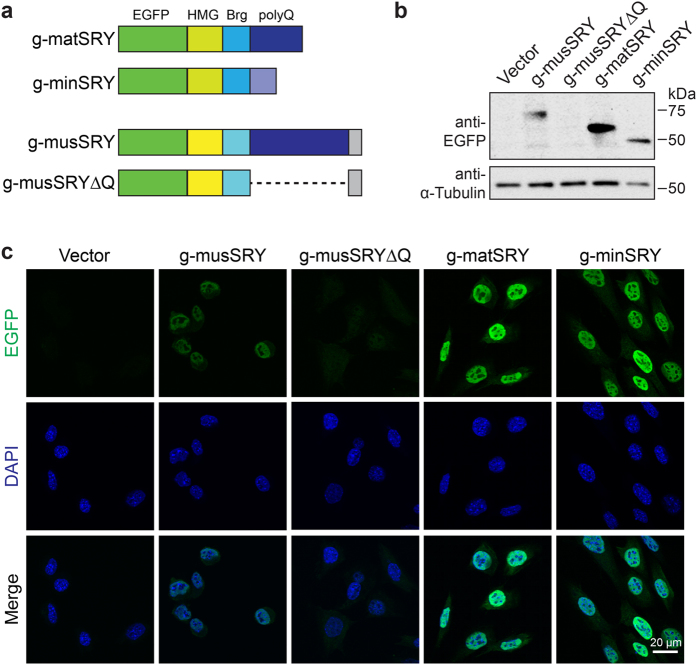
The degraded polyQ tract in *M. minutoides* SRY does not compromise protein stability. (**a**) Schematic of EGFP-tagged matSRY and minSRY proteins (g-matSRY and g-minSRY, respectively). EGFP-tagged musSRY (g-musSRY) and musSRY lacking the polyQ domain (g-musSRYΔQ) are also shown here for comparison. (**b**) Both g-minSRY and g-matSRY were detected in 15P-1 stable cell lines by Western blotting using an anti–EGFP antibody. g-musSRY and g-musSRYΔQ were included as positive and negative controls, respectively. Predicted molecular weight: g-musSRY, 77.1 kDa; g-musSRY ΔQ, 47.9 kDa; g-minSRY, 50.7 kDa; g-matSRY, 59.9 kDa. A blot using anti–α-Tubulin served as loading control. Full-length blots are presented in Supplementary Fig. S15. (**c**) Both g-matSRY and g-minSRY were detected in the nuclei of 15P-1 cells by immunofluorescence using an anti–EGFP antibody. g-musSRY and g-musSRYΔQ were included as positive and negative controls, respectively.

We have previously established that the polyQ tract is essential for protein stabilization and transactivation by SRY in *M. musculus*^11^. Because minSRY has a highly degraded polyQ tract, we examined whether the stability and transactivation potential of minSRY is compromised. We found that, unlike a EGFP-tagged *M. musculus* SRY mutant protein completely lacking the polyQ tract that is barely detectable when stably expressed in mouse Sertoli-like 15P-1 cell line^11^, EGFP-tagged min- and matSRY protein (Fig. 2a) were readily detected by Western blot and immunofluorescence (Fig. 2b,c) in stable 15P-1 cell lines, indicating that the stability of minSRY is not affected by its degraded polyQ tract.

**Figure 3:**
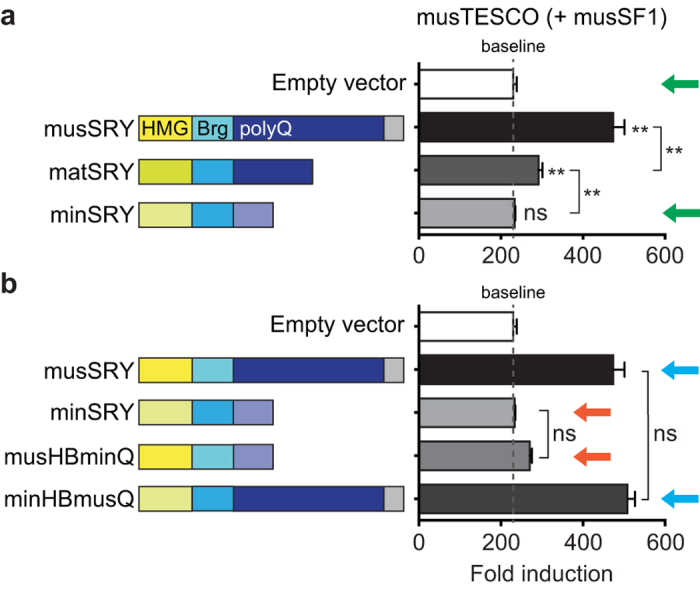
*M. minutoides* SRY fails to activate the *M. musculus* TESCO reporter due to the degeneration of its C-terminal polyQ tract. (**a**) minSRY, unlike musSRY and matSRY, failed to synergize with musSF1 to activate a luciferase construct containing *M. musculus* TESCO (compare green arrows). (**b**) Similar to minSRY, the musHBminQ mutant failed to activate musTESCO-Luc in the presence of musSF1 (compare orange arrows), whereas the mutant minHBmusQ restored the transactivation ability to that of musSRY (compare blue arrows). The luciferase activity of musTESCO co-transfected with the empty vector in the absence of SF1 was set to 1. SRY constructs do not activate TESCO in the absence of SF1, and thus the −SF1 data essentially showed unchanged base level activities of the musTESCO-Luc reporter. Therefore, for simplicity, only the + SF1 data are presented here, as mean ± s.e.m (n = 3). Dashed lines indicate the level of baseline (empty vector + SF1). (**) *P* < 0.01 vs. empty vector, or as indicated, one-way repeated measures ANOVA with Holm-Sidak multiple comparisons test. ns, not significant.

In mouse sex determination, the 1.4-kb TESCO enhancer element plays a significant role in mediating the induction of *Sox9* expression by SRY in the presence of SF1^5^, although it is likely that other, yet to be identified testis-specific *Sox9*-enhancer elements may also contribute to SRY’s regulation of *Sox9*. We have previously shown that the ability of a series of SRY mutant proteins to transactivate a *M. musculus* TESCO-luciferase reporter construct (musTESCO-Luc)^5^ in the heterologous cell line HEK293 correlates closely with their ability to induce *Sox9* expression and direct male sex determination in transgenic mouse embryos^11^. We therefore examined the ability of min- or matSRY protein to activate the musTESCO-Luc reporter in the presence of *M. musculus* SF1 (musSF1) in HEK293 cells. We observed activation by matSRY, albeit weaker than musSRY (Fig. 3a), consistent with our previous observation that, compared with musSRY, *M. domesticus* SRY exhibits reduced TESCO activation due to its shortened polyQ tract^11^. Strikingly, minSRY failed to activate musTESCO-Luc at all in this system (Fig. 3a, compare green arrows).

The failure of minSRY to activate musTESCO-Luc could be caused by either its intrinsic structural changes or incompatibility between minSRY and musSF1. We therefore investigated whether potential sequence variations between *M. musculus* and *M. minutoides Sf1* could account for the loss of musTESCO activation by minSRY. To this end, we sequenced coding exons 2–7 of *Sf1* gene in *M. minutoides* and *M. mattheyi* (Supplementary Fig. S7) and found four and one amino acid substitutions in *M. minutoides* and *M. mattheyi* SF1 (min- and matSF1) respectively, compared with musSF1 (Supplementary Figs. S8 and S9a). Nevertheless, these sequence changes had no measurable effect on SF1’s ability to synergize with SRY to activate musTESCO (Supplementary Fig. S9b,c). Importantly, minSRY failed to activate the musTESCO-Luc reporter in the presence of either mat- or minSF1. These results indicate that the sequence changes in mat/minSF1 do not significantly alter their ability to synergize with SRY to activate TESCO (at least in these *in vitro* reporter assays), and that the loss of transactivation ability of minSRY is most likely caused by its intrinsic structural changes.

We reasoned that either the degraded polyQ domain of minSRY had lost transactivation potential, or variations in the HMG + bridge domains (Supplementary Fig. S6)^14^ impaired binding to TESCO and/or interaction with SF1. To distinguish these possibilities, we generated two mutant constructs and analyzed their ability to activate musTESCO-Luc in the presence of musSF1. The mutant musHBminQ—combining the musSRY HMG + bridge domains with the *M. minutoides* polyQ tract—failed to activate musTESCO-Luc, similar to minSRY (Fig. 3b, compare orange arrows). In contrast, the mutant minHBmusQ—combining the minSRY HMG + bridge domains with the *M. musculus* polyQ tract—showed fully restored ability to activate musTESCO-Luc (Fig. 3b, compare blue arrows). The use of mat/min SF1 appeared to have no effect on the activities of these SRY mutants (Supplementary Fig. S9b,c). Thus, the abolished transactivation capacity of minSRY on *M. musculus* TESCO is primarily caused by the loss of a typical polyQ tract.

### *M. minutoides* and *M. mattheyi* TESCO are severely debilitated

In *M. musculus*, the TESCO enhancer acts as a regulatory hub for *Sox9* expression^5^,^20^,^21^,^22^. Debilitation of the SRY-*Sox9* nexus due to various sequence changes within TESCO may have led to the emergence of *Sry*-independent sex-determining systems in the mole voles *Ellobius lutescens* and *E. tancrei*^23^, and the Japanese spiny rats *Tokudaia osimensis* and *T. tokunoshimensis*^24^. We therefore analyzed TESCO sequences in *M. minutoides* and *M. mattheyi* (minTESCO and matTESCO) to look for structural irregularities that might similarly disrupt this nexus.

**Figure 4:**
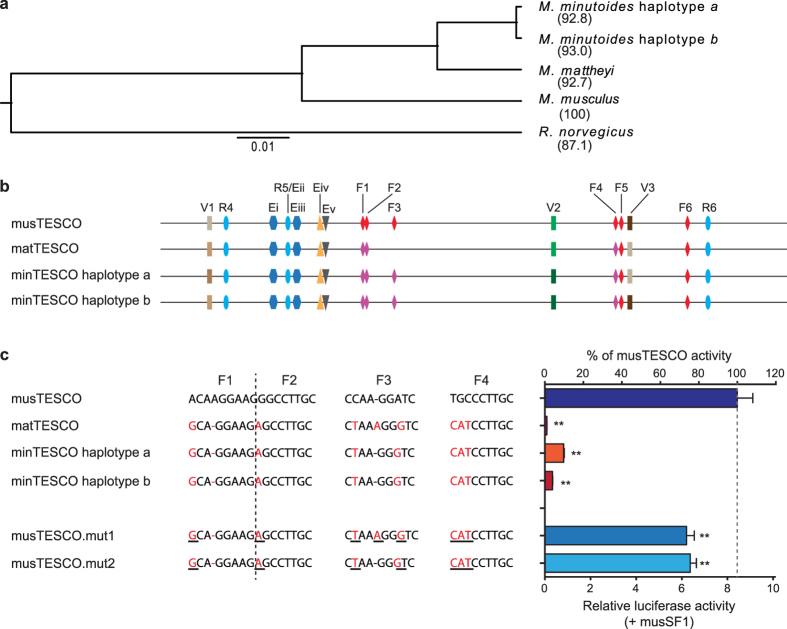
Markedly reduced enhancer activity of TESCO in *M. mattheyi* and *M. minutoides*. (**a**) Maximum likelihood phylogeny using TESCO sequences. Numbers indicate percent sequence identity scores compared to musTESCO. (**b**) Schematic of TESCO enhancers from three *Mus* species. R4-6, SRY binding sites; F1-6, SF1 binding sites; Ei-v, evolutionarily conserved regions; V1-3, sequence variations between minTESCO a and b. Sequence changes at sites F1-4 may impair SF1 binding and are indicated with different colours. The F3 site predicted to have SF1 binding disrupted is removed from the matTESCO diagram. (**c**) Sequence comparison of F1-4 sites from the three species, and the mutants musTESCO.mut1/2. Sequence changes compared with musTESCO are in red and mutated sequences are underlined. Compared with musTESCO, mat/minTESCO showed markedly reduced activities in the presence of musSF1. Mutations of SF1 binding sites F1-4 in musTESCO to the corresponding sequence in matTESCO (musTESCO.mut1) or minTESCO (musTESCO.mut2) caused mildly reduced reporter activities in the presence of musSF1. Data are presented as TESCO luciferase activity normalized to co-transfected CMV-renilla luciferase activity. Error bars: s.e.m. (*n* = 3). Dashed lines indicate the levels of musTESCO activity. (**) *P* < 0.01 vs. musTESCO, one-way repeated measures ANOVA with Dunnett’s multiple comparisons test.

**Figure 5:**
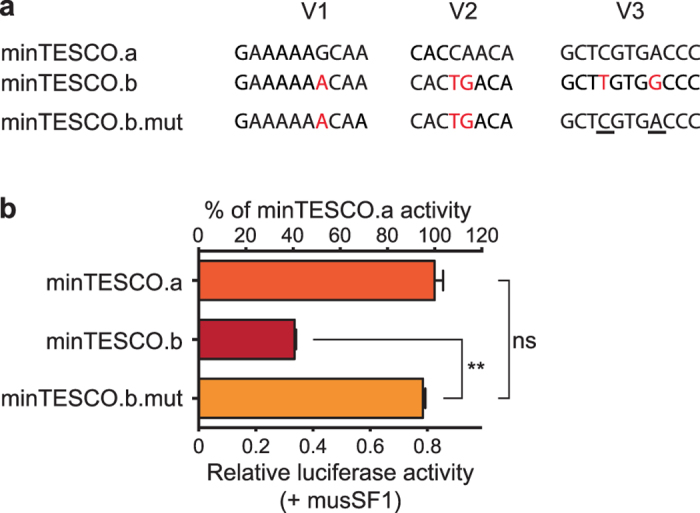
Sequence variations at the V3 site between two haplotypes of *M. minutoides* TESCO results in reduced enhancer activity. (**a**) Sequence comparison of V1-3 between minTESCO haplotypes a and b. Sequence changes between the two haplotypes are in red. Mutated bases in minTESCO.b.mut are underlined. (**b**) Compared with minTESCO.a, minTESCO.b had its transcriptional activity (in the presence of musSF1) halved. minTESCO.b.mut with V3 site mutated to the corresponding sequence in minTESCO.a showed fully restored reporter activity in the presence of musSF1. Data are presented as TESCO luciferase activity normalized to co-transfected CMV-renilla luciferase activity. Error bars: s.e.m. (*n* = 3). (**) *P* < 0.01, one-way repeated measures ANOVA with Dunnett’s multiple comparisons test. ns, not significant.

matTESCO and minTESCO have sequence identities of approximately 93% with musTESCO (Fig. 4a). Most sequence variations lie outside regulatory elements previously identified in *M. musculus*, including the SRY binding sites R4-R6^5^ and the evolutionarily conserved regions ECRi-v^25^ (Supplementary Fig. S10). Notably, sequence variations were identified in both mat- and minTESCO at SF1 binding sites F1-F4^5^ (Fig. 4b,c) which may impair SF1 binding^26^,^27^. Sequences at SF1 binding sites F5-F6 in mat/minTESCO remain identical to those in musTESCO (Supplementary Fig. S10). Moreover, two haplotypes minTESCO.a and -.b differ at three sites (V1-3; Figs 4b and 5a).

We next examined the transcriptional activities conferred by mat/minTESCO enhancers using luciferase reporter assays in HEK293 cells. The transcriptional activities of mat/minTESCO in the presence of musSF1 decreased significantly compared to musTESCO (Fig. 4c), due to a severely diminished response to musSF1: fold induction by musSF1 (that is, the ratio of activity in the presence:absence of SF1) fell to approximately 8 or 24% in the case of matTESCO and minTESCO.b respectively (Supplementary Fig. S11). As a result, minTESCO.a retained ~10% of musTESCO activity, while the activities of matTESCO and minTESCO.b were 1–3% of that of musTESCO (Fig. 4c).

The severely decreased ability of mat/minTESCO to respond to SF1 may be a direct consequence of the sequence variations at known SF1 binding sites F1-4 (Fig. 4b,c). We therefore mutated these sites in musTESCO-Luc to the corresponding sequences in mat/minTESCO (musTESCO.mut1/2; Fig. 4c). Surprisingly, the two TESCO mutants showed only mildly reduced reporter activities (Fig. 4c) and response to musSF1 (Supplementary Fig. S11), nowhere near those of mat/minTESCO, suggesting the likely presence of unidentified SF1 binding sites within TESCO. Supporting this view, mutation of the V3 site in minTESCO.b to the corresponding motif found in minTESCO.a (minTESCO.b.mut; Fig. 5a), fully restored its activity and response to musSF1 to the levels of minTESCO.a (Fig. 5b and Supplementary Fig. S12). Similar results were obtained with mat- and minSF1 (Supplementary Fig. S13 and data not shown).

Together, these results demonstrate that TESCO enhancers in *M. minutoides* and *M. mattheyi* are barely functional, compared to *M. musculus* TESCO. Moreover, our data strongly indicate that sequences outside the previously identified SF1 (and SRY) binding sites may play hitherto unappreciated roles in modulating TESCO activity and *Sox9* expression in *Mus* species.

### *M. minutoides* SRY activates its cognate TESCO in the presence of SF1

**Figure 6:**
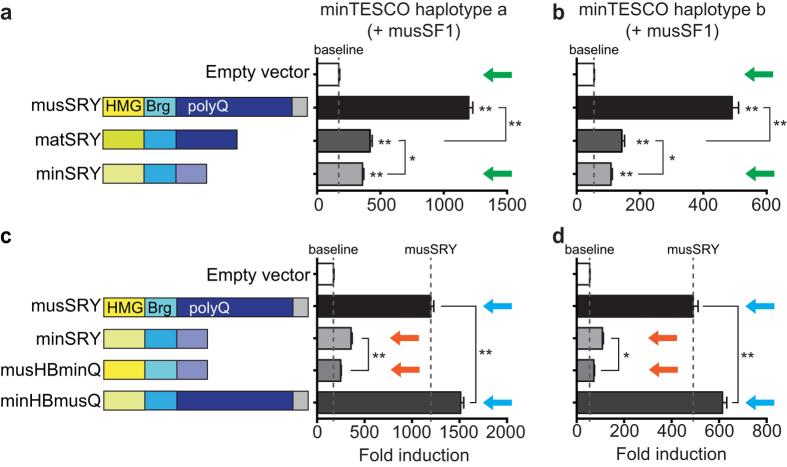
Co-evolution of *Sry* and TESCO in *M. minutoides*. (**a**,**b**) minSRY activated minTESCO.a/b in the presence of musSF1 (compare green arrows), albeit more weakly than musSRY and matSRY. (**c**,**d**) minSRY significantly outperformed musHBminQ in activating both minTESCO.a/b reporters (compare orange arrows). Conversely, minHBmusQ mutant significantly outperformed musSRY (compare blue arrows). The luciferase activity of each luciferase reporter co-transfected with the empty vector in the absence of SF1 was set to 1. SRY constructs do not activate TESCO in the absence of SF1, and thus the −SF1 data essentially showed unchanged base level activities of TESCO-Luc reporter. Therefore, for simplicity, only the + SF1 data are presented here as mean ± s.e.m (n = 3). Dashed lines indicate the levels of baseline (empty vector + musSF1) or synergistic activation by musSRY + musSF1. (*) *P* < 0.05, (**) *P* < 0.01 vs. empty vector, or as indicated, one-way repeated measures ANOVA with Holm-Sidak multiple comparisons test. ns, not significant.

The failure of minSRY to activate musTESCO (Fig. 3a) may indicate that, in *M. minutoides*, SRY is no longer functioning as the testis-determining gene and a newly evolved Y-linked testis-determining gene may be operative. Alternatively, TESCO and SRY may have co-evolved in this species, such that minSRY remains able to activate its cognate TESCO. To distinguish these possibilities, we performed luciferase reporter assays in HEK293 cells testing synergistic activation of min/matTESCO luciferase reporters by various combinations of SRY and SF1 constructs. We found that, in the presence of musSF1, minSRY robustly activated its cognate minTESCO.a/b (Fig. 6a,b, compare green arrows). However, the fold induction by minSRY was much weaker compared to musSRY which has a longer polyQ tract (Fig. 6a,b). minSRY also activated matTESCO (Supplementary Fig. S14c), despite its lack of activity on musTESCO (Fig. 3a). Similar results were obtained with mat/minSF1 constructs (Supplementary Fig. S14). Thus, *Sry* and TESCO appear to have co-evolved to retain functional compatibility in *M. minutoides*.

We reasoned that that mat/minTESCO may have acquired sequence changes making them more responsive to SRY, thus allowing a weaker SRY such as minSRY to function. Consistent with this hypothesis, minTESCO was activated > 7 fold by musSRY in the presence of SF1 (Fig. 6a,b), while musTESCO was only activated ~2 fold by musSRY (Fig. 3a).

Furthermore, minSRY may have also acquired adaptive changes, presumably within the HMG box and bridge domains, since its degraded polyQ domain represents a much weaker transactivation domain compared with its counterpart in musSRY (Fig. 3b), and is unlikely to offer any adaptive advantages. To test this possibility, we made various combinations of the HMG (H), bridge (B) and polyQ-domains (Q), and assayed their ability to activate both minTESCO.a/b luciferase reporters. Consistent with our hypothesis, minSRY outperformed the musHBminQ mutant, and the minHBmusQ mutant eclipsed musSRY (Fig. 6c,d, compare orange and blue arrows respectively).

These results support the notion that *Sry* and TESCO may have co-evolved in *M. minutoides*, and further indicate that the HMG box and bridge domains of SRY may have a mild impact on TESCO activation. This conclusion is in accordance with a previous report that *M. musculus* polyQ tract, as a strong transactivation domain, allows and compensates for otherwise deleterious amino acid substitutions in the HMG box^28^.

Taken together, our results indicate that the SRY/SF1/TESCO nexus has been severely weakened in *M. minutoides* through two different mechanisms. *M. minutoides* SRY’s capacity to activate TESCO is severely compromised primarily due to its degraded polyQ tract, as SRY binding sites within TESCO remain intact. Secondly, SF1 protein in *M. minutoides* possesses comparable activity compared with *M. musculus* SF1, but DNA sequence changes in its target sites in TESCO cause a significantly reduced response.

## Discussion

In contrast to the high plasticity seen in many vertebrate classes, sex determination in mammals is relatively static and almost invariably based on an XX female/XY male system. Exceptions are rare^29^, and afford the potential to gain insights into the evolution of sex-determining systems as well as into mechanistic issues such as the interplay between testis- and ovarian-determining pathways. Such is the case with the unusual X*Y sex reversal system found in *M. minutoides*, the focus of the present study. By studying critical elements of the testis-determining pathway, we have shown that the activities of *M. minutoides* SRY, SF1 and the *Sox9* enhancer TESCO are compromised but still capable of functioning such that they are able to generate XY males. Assuming our *in vitro* data broadly reflect the activities of these elements *in vivo*, the phenomenon of X*Y sex reversal in this species is likely to be due to a combination of an as-yet unidentified variant gene(s) on the X* chromosome and a weakening of the male sex-determining pathway.

### Co-evolution of SRY and TESCO in *M. minutoides*

Our analyses reveal that *M. minutoides* SRY has completely lost its ability to activate TESCO from other species, and that *M. minutoides* TESCO also has degenerated so that it retains only very limited ability to respond to SF1, compared with its counterpart in *M. musculus*. However, *M. minutoides* SRY is clearly able to activate its cognate TESCO, albeit weakly, indicating that SRY and its target TESCO enhancer may have co-evolved in this species to secure male sex determination.

TESCO in *M. mattheyi* and *M. minutoides* may have acquired sequence changes outside the previously identified SRY binding sites R4-R6 to enable itself to respond more strongly to SRY, thus compensating for the weaker transactivating capacity of SRY in these species. In addition, SRY itself has also evolved. It has been suggested that *Sry* is under positive selection^9^,^30^,^31^. Supporting this view, we found several amino acid changes in the sequences of the HMG and bridge domains of matSRY and minSRY. These adaptive sequence changes in TESCO and SRY may improve the binding affinity of SRY to TESCO and/or the interaction between SRY and SF1. In this regard, we note an arginine to cysteine change at amino acid 17 (R17C) in the HMG box (Supplementary Fig. S6), a site involved in electrostatic and hydrophobic interactions between SRY and its target DNA^32^.

Despite the co-evolution of SRY and TESCO in *M. minutoides*, the absolute activity of the SRY/SF1/TESCO nexus in this species is reduced to less than 10% of that in *M. musculus*, due to the severely impaired transactivating capacity of SRY and reduced response of TESCO to SF1.

### Interaction of the X* chromosome with the weakened male sex-determining pathway

Even though the *Sry* pathway is weakened in *M. minutoides*, it is able to function in the presence of a normal X but not in the presence of an X*. We hypothesize that the X* chromosome carries a stronger pro-ovarian allele, a stronger anti-testis allele, or a weaker allele essential for testis-determining signalling, compared to a normal X. We further hypothesize that this X*-linked modifier gene favours ovarian development but does not cause XY sex reversal in combination with a strong *Sry* allele such as those in *M. musculus* or *M. mattheyi*. Only when combined with a weakened male sex-determining pathway such as that found in *M. minutoides* does XY femaleness result.

A similar scenario has been described previously in laboratory mice *M. musculus*, where additional copies of the X-linked *Dax1* gene in transgenic mice do not affect normal testis development in the presence of an *M. musculus* Y chromosome, but cause ovarian or ovotestis development^33^ when combined with an *M. domesticus poschiavinus* Y chromosome carrying a weak *Sry* allele^34^. Nevertheless, *Dax1* does not seem to be involved in X*Y sex reversal in *M. minutoides*, as the X*-linked *Dax1* copy shows no sign of changes in either copy number or expression^13^. Therefore, a true test of the hypothesis that the X* harbours a pro-ovarian modifier gene rests on identification of suitable candidates for such a role and testing their activity in different mouse strains.

### Origin and fixation of X* chromosome

**Figure 7:**
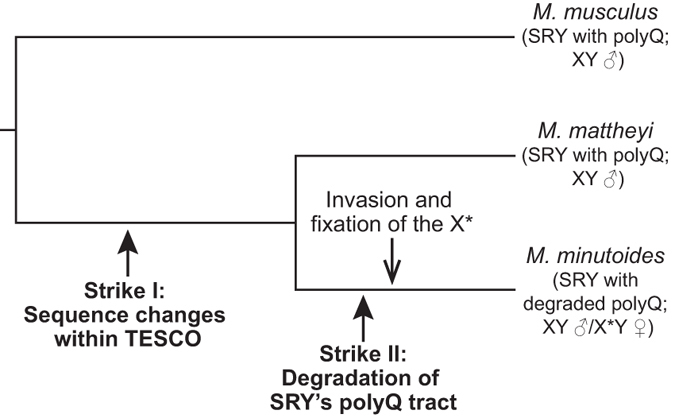
A model for the evolution of the atypical sex determination system in *M. minutoides*. In a common ancestor to *M. mattheyi* and *M. minutoides*, multiple sequence variations within TESCO occurred. This first strike significantly reduced the basal transcriptional activity of TESCO and attenuated its response to SF1. Subsequently, a second strike hit *Sry* in an ancestor to *M. minutoides*, causing a severe degradation of its polyQ tract. Such a degenerated SRY retains only weak capacity to activate TESCO and *Sox9* expression. Together, these genetic events have rendered the male sex-determining pathway vulnerable in *M. minutoides*, which may have facilitated the invasion and subsequent spread of the X* chromosome (and feminising modifier(s) thereon). The presence of X* chromosome in X*Y individuals overwrites the fragile male sex-determining pathway and leads to female sex reversal.

The finding that the *Sry*-driven male pathway is compromised to some extent in *M. minutoides* logically suggests that it is the combination of a neomorphic gene on the X* chromosome and the weakened *Sry* pathway that underpin the X*Y sex reversal in *M. minutoides.* How might this unusual sex-determination system have arisen? Our findings lead us to propose the model illustrated in Fig. 7. In this model, in a common ancestor to *M. mattheyi* and *M. minutoides*, TESCO acquired sequence variations that caused both decreased basal activity and reduced response to SF1. Subsequently, in an ancestor to *M. minutoides*, the SRY polyQ tract degenerated further, likely due to the rapid sequence evolution and degradation of the Y chromosome-linked genes and/or the CAG microsatellite instability^30^,^35^, causing diminished capacity to activate TESCO. With the activity of both SRY and its key target TESCO being compromised, the male pathway in *M. minutoides* became vulnerable to the influence of a modifier gene. It is conceivable that a subsequent chromosomal rearrangement event of the ancestral X created a tight linkage between this modifier and genes conferring enhanced reproductive outcome, thus generating the X*. In the presence of a weakened *M. minutoides* type *Sry* allele, the X*-borne modifier caused ovarian development, resulting in X*Y females with high fecundity and a skewed sex ratio.

In contrast to laboratory mouse models where sex reversed XY females are almost always sterile^36^, X*Y females in *M. minutoides* show enhanced reproductive performance compared with the XX and XX*^37^. Increased fecundity of X*Y over XX females has also been reported in several other rodent species with sex determination systems similar to that in *M. minutoides*, including the lemming *Myopus schisticolor* and *Dicrostonyx torquatus*^38^,^39^, and the South American grass mouse *Akodon azarae*^40^,^41^.

Another common feature of species with X*Y females is an expected female-biased sex ratio^38^,^41^, which has been suggested to contribute to the invasion and maintenance of the X* in certain circumstances^42^,^43^,^44^. Hence it is possible that the X* subsequently was stabilized in the population due to the reproductive advantage of X*Y females over XX and XX* and/or a selection for favouring a female-biased sex ratio or for restoring a 1:1 sex ratio after the invasion of a Y chromosome distorter^43^,^44^,^45^,^46^.

### Implications for evolution of mammalian sex-determining systems independent of *Sry*

From the time when *Sry* first arose as a variant of the X-chromosomal gene *Sox3*^47^,^48^,^49^ and became the male sex determinant in a mammalian ancestor, most genes on the neo-Y chromosome began an inexorable process of loss or pseudogenization, thus resulting in the highly evolved and decayed Y chromosomes currently found in mammals^50^,^51^,^52^,^53^. Our data show that *Sry* function has deteriorated in *M. minutoides*, likely due to ongoing degradation of *Sry* in African pygmy mice. With the activities of both SRY and its target TESCO being compromised, the testis-determining pathway in *M. minutoides* is likely operating at a threshold level.

The current situation in *M. minutoides* may represent an intermediate state in which both *Sry*-dependent testis-determination and an X*-dependent dominant feminizing mechanism are in operation. With the further passage of evolutionary time, SRY’s polyQ tract may undergo further degradation in *M. minutoides*, as has already occurred in some identified *Sry* haplotypes. With the translocation of Y-linked essential male fertility genes to other chromosomes^54^,^55^, Y chromosome would no longer be required and would eventually be lost, supplanted by a neo-Y chromosome (and a new male sex-determining gene that should overpower the weakened *Sry*) or replaced by a XO/X*O sex determination system with a masculinizing X and a feminizing X* chromosome, as has likely occurred in the Japanese spiny rats *T. osimensis* and *T. tokunoshimensis*^56^,^57^ and the mole vole *E. lutescens*^58^,^59^.

In this regard, another species of African pygmy mice, *M. triton*, has lost the Y chromosome, with both males and females having an XO karyotype^60^ and may represent this ultimate evolutionary step. Undoubtedly, more examples remain to be discovered. Further study of these exceptions to the rule, in particular the identification of the X*-borne modifier gene(s) that cause X*Y sex reversal and genes that trigger sex development in the absence of *Sry*, will illuminate the normal process of sex development and identify new candidate genes whose loss of function might cause human disorders of sex development.

## Methods

### PCR amplification, cloning and Sanger sequencing

No live animals were used in this study. Genomic DNA was extracted from tissue of an XY individual of *M. minutoides* or *M. mattheyi* (specimens collected under permits 2003/PFHG/05/GUI, 1155 MDCS/CAB-1/kss^14^) using a Qiagen DNeasy kit and subject to PCR amplification (Takara LA PCR kit) with primers designed based on *M. musculus* sequence. Primer sequences are provided in Supplementary Table S1. Eight (for *Sry*), four (for TESCO), or three (for each *Sf1* coding exon) independent clones from each PCR product were sequenced. Sequences were aligned using ClustalW^61^. Identity scores were calculated using GeneStream II^62^.

### Phylogenetic reconstruction using TESCO sequence

TESCO sequences in *M. musculus* and *Rattus norvegicus* were retrieved from mm10 or rn6 reference genome, respectively. The maximum likelihood phylogeny was reconstructed using BEAST 2.0^63^ and visualised using FigTree. *R. norvegicus* was included as an outgroup.

### Expression analyses of SRY protein in stable mouse Sertoli-like 15P-1 cell lines

The *M. mattheyi* or *M. minutoides Sry* coding region with a preceding EGFP coding sequence inserted in frame was subcloned into pMIH retroviral vector^64^. Stable 15P-1 cell lines were established by infection of individual retrovirus produced as described^11^,^65^. Western blot and immunofluorescence analyses were performed as described^11^ with anti–EGFP (Abcam, Ab5450) or anti–α-Tubulin (Sigma, T5168) antibodies.

### Plasmids and luciferase reporter assays

Chimeric mutants musHBminQ and minHBmusQ in pMIH vector were generated using Quikchange method (Agilent). To generate TESCO luciferase reporter constructs, *M. musculus* TESCO in pTESCO-δ51-LucII (musTESCO-Luc)^5^ was replaced with *M. mattheyi* or *M. minutoides* TESCO (mat/minTESCO-Luc). Mutagenesis of SF1 binding sites in musTESCO-Luc or the V3 site in minTESCO.b-Luc was carried out using Quikchange method (Agilent).

pcDNA-musSf1 plasmid containing *M. musculus Sf1* coding sequence has been described previously^66^. pcDNA-matSf1 was generated by replacing the EcoRI-BstXI fragment of pcDNA-musSf1 with a chemically synthesized DNA fragment (Integrated DNA technologies) containing the *M. mattheyi*-specific non-synonymous nucleotide change. To make pcDNA-minSf1, *M. minutoides Sf1* coding region was chemically synthesized (Integrated DNA technologies) and subsequently cloned into pcDNA3.1( + ) (Life Technologies).

Luciferase reporter assays were conducted as described^11^. Briefly, HEK293 cells were co-transfected with a TESCO luciferase construct, a pMIH empty vector or a pMIH construct containing various *Sry* or *Sry* mutant sequences, and an empty pcDNA3 or a pcDNA3 construct containing various *Sf1* sequence. A cytomegalovirus (CMV)-renilla luciferase plasmid^11^ was included as a control for transfection efficiency. Multiplicity-adjusted *P* values were calculated using GraphPad Prism 6.

## Additional Information

**Accession codes:** DNA sequences were deposited into GenBank under the accession numbers KP063038-KP063042 (M. minutoides Sry), KP063043-KP063049 (M. mattheyi Sry), KP063050-KP063052 (M. minutoides and M. mattheyi TESCO), and KT340072-KT340073 (M. minutoides and M. mattheyi Sf1).

**How to cite this article**: Zhao, L. *et al*. Reduced Activity of SRY and its Target Enhancer *Sox9*-TESCO in a Mouse Species with X*Y Sex Reversal. *Sci. Rep.* **7**, 41378; doi: 10.1038/srep41378 (2017).

**Publisher's note:** Springer Nature remains neutral with regard to jurisdictional claims in published maps and institutional affiliations.

## Supplementary Material

Supplementary Information

## Figures and Tables

**Table 1:** Multiple *Sry* copies are present in *M. minutoides* and *M. mattheyi*.

Clone #	Haplotype	*Sry* DNA sequence variation	SRY protein sequence variation
*M. minutoides*			
1	a	c.514_534del	p.(E170_Q176del) in the polyQ domain
2	a	c.514_534del	p.(E170_Q176del) in the polyQ domain
3	b	c.[352A > G; 613_615del]	p.(T118A) in the bridge domain
4	c	c.[51T > C; 410A > G; 573T > C]	p.(D137G) in the polyQ domain
5	d	Same as the consensus	Same as the consensus
6	e	c.316A > G	p.(R106G) in the bridge domain
7^a^	d	Same as the consensus	Same as the consensus
8	d	Same as the consensus	Same as the consensus
*M. mattheyi*			
1	a	c.971_973del	Same as the consensus
2	b	c.562_564del	p.(Q188del) in the polyQ domain
3	c	c.503_574del	p.(Q172_Q195del) in the polyQ domain
4^a^	d	Same as the consensus	Same as the consensus
5	b	c.562_564del	p.(Q188del) in the polyQ domain
6	e	c.503A > G	p.(H168R) in the polyQ domain
7	f	c.787C > T	Same as the consensus
8	g	c.451C > T	p.(Q150*); almost complete truncation of the polyQ domain

^a^Reference clones used in cross-species sequence comparisons and subsequent experimental analyses.
