# Supplementary material for: Reduced Activity of SRY and its Target Enhancer Sox9-TESCO in a Mouse Species with X*Y Sex Reversal
Source: Sci Rep. 2017 Feb 3;7:41378. doi: 10.1038/srep41378 (PMC5290746; doi:10.1038/srep41378)
Supplement: Supplementary Information [file srep41378-s1.pdf]

Supplementary Information

Reduced Activity of SRY and its Target Enhancer *Sox9*-TESCO in a Mouse Species with X*Y Sex Reversal

Liang Zhao[1], Alexander Quinn[1], Ee Ting Ng[1], Frederic Veyrunes[2] and Peter Koopman[1]*

1. Institute for Molecular Bioscience, The University of Queensland, Brisbane, QLD 4072, Australia

2. Institut des Sciences de l'Evolution de Montpellier, Université Montpellier II, CNRS, Montpellier, France

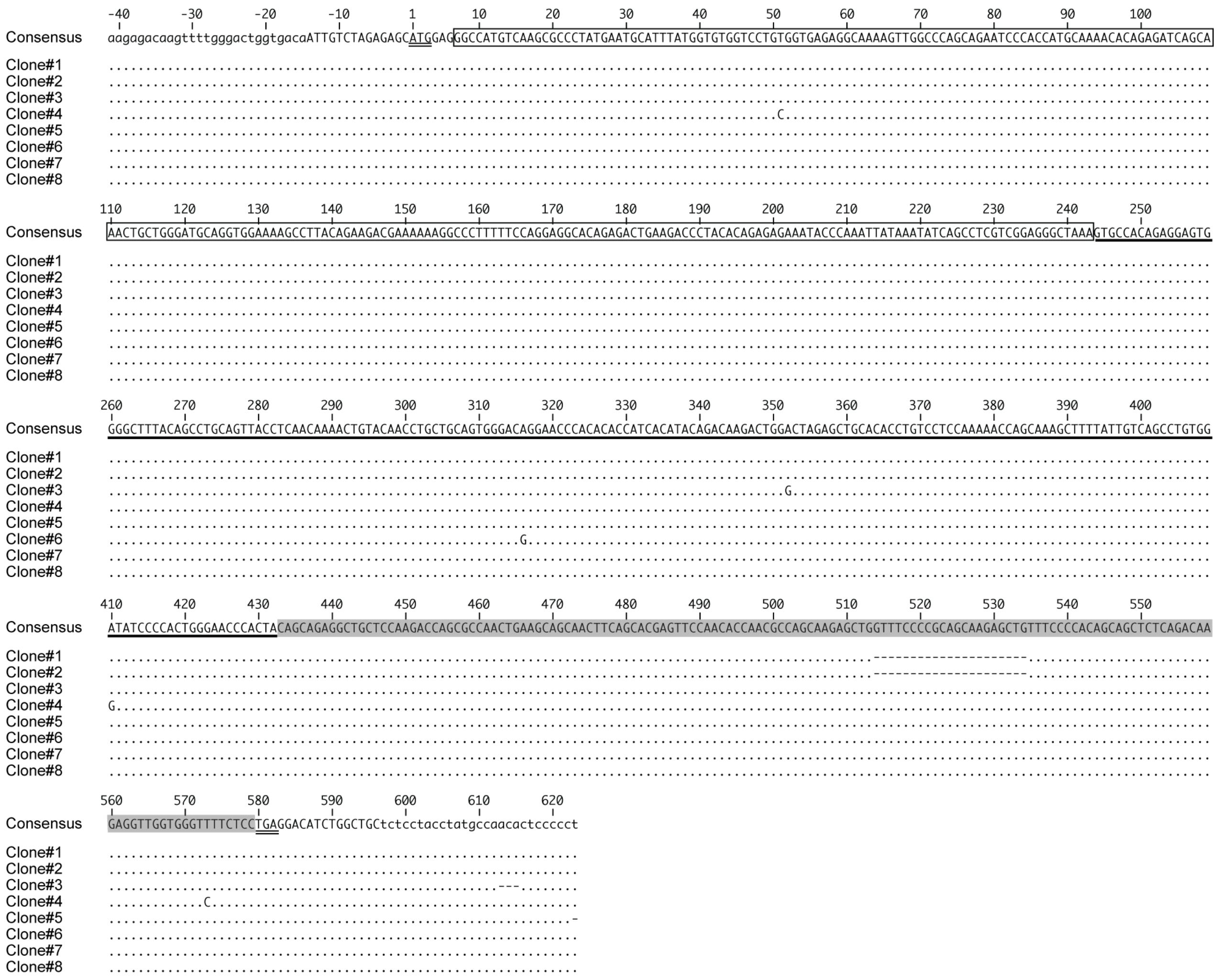

Supplementary Figure S1. Nucleotide sequence alignment of eight independent *M. minutoides Sry* clones. Five haplotypes were identified (see also Table 1). The consensus sequence is displayed above the alignment. Sequences identical to the consensus are indicated by dots, while missing nucleotides are depicted by hyphens. Binding sites of PCR primers are in lower case. Start and stop codons are double-underlined. Sequences encoding the HMG, Bridge, and polyQ domains are boxed, underlined or in grey, respectively.

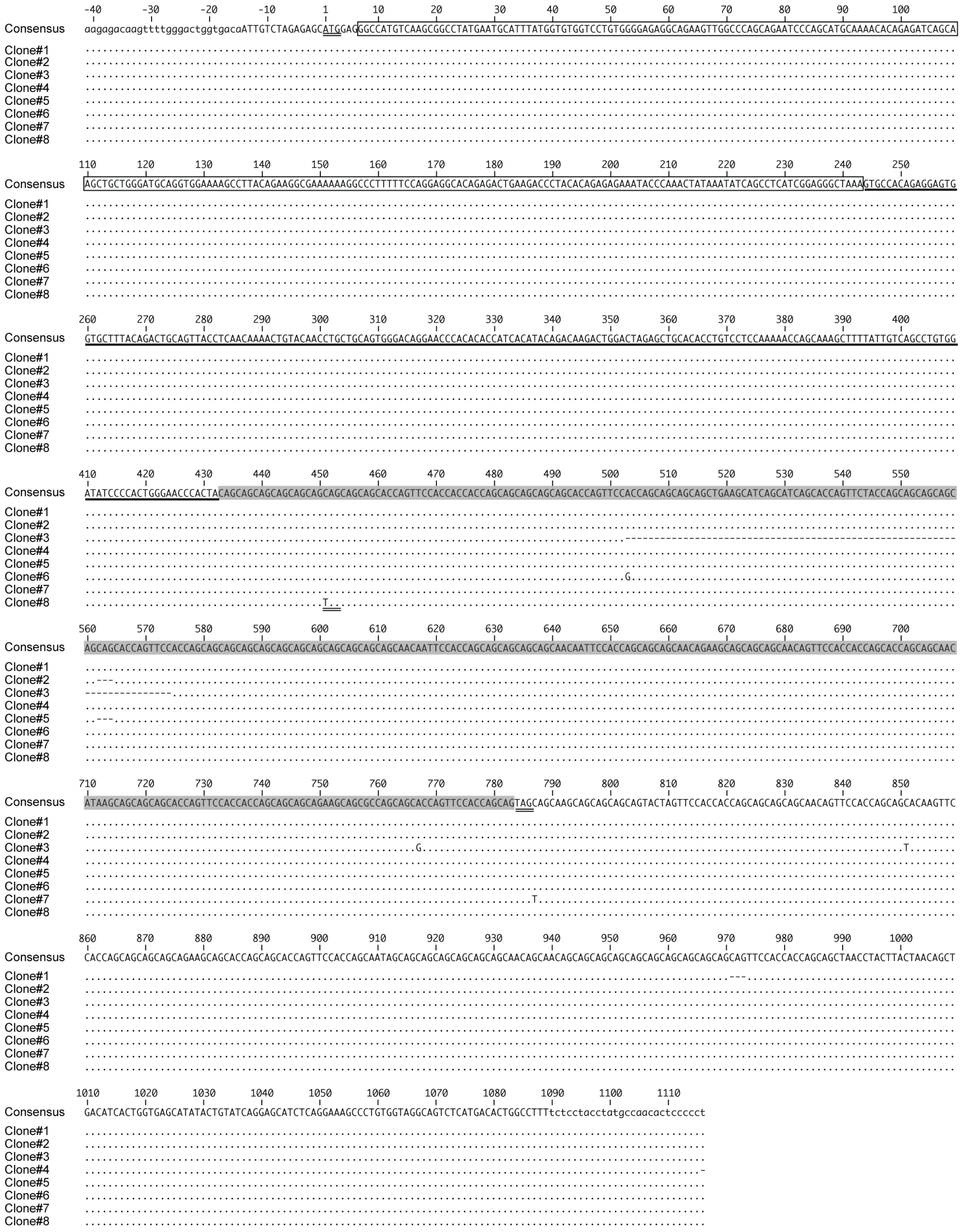

Supplementary Figure S2. Nucleotide sequence alignment of eight independent *M. mattheyi* *Sry* clones. Seven haplotypes were identified (see also Table 1). The consensus sequence is displayed above the alignment. Sequences identical to the consensus are indicated by dots, while

missing nucleotides are depicted by hyphens. Binding sites of PCR primers are in lower case. Start and stop codons are double-underlined. Sequences encoding the HMG, Bridge, and polyQ domains are boxed, underlined or in grey, respectively.

```
                   10        20        30        40        50        60        70        80        90       100
Consensus  MEGHVKRPMNAFMVWSCGERQKLAQQNPTMQNTEISKLLGCRWKSLTEDEKRPFFQEAQRLKTLHREKYPNYKYQPRRRAKVPQRSGALQPAVTSTKLYN
Clone#1    ....................................................................................................
Clone#2    ....................................................................................................
Clone#3    ....................................................................................................
Clone#4    ....................................................................................................
Clone#5    ....................................................................................................
Clone#6    ....................................................................................................
Clone#7    ....................................................................................................
Clone#8    ....................................................................................................

                  110       120       130       140       150       160       170       180       190
Consensus  LLQWDRNPHTITYRQDWTRAAHLSSKNQQSFYCQPVDIPTGNPLQQRLLQDQRQLKQQLQHEFQHQRQQELVSPQQELFPHSSSQTRGWWVFS
Clone#1    .....................................................................-------.................
Clone#2    .....................................................................-------.................
Clone#3    .................A...........................................................................
Clone#4    ....................................G........................................................
Clone#5    .............................................................................................
Clone#6    .....G.......................................................................................
Clone#7    .............................................................................................
Clone#8    .............................................................................................
```

Supplementary Figure S3. Alignment of the deduced SRY peptide sequences in *M. minutoides*. The consensus sequence is displayed above the alignment. Residues identical to the consensus are indicated by dots, while missing residues are depicted by hyphens. The HMG, Bridge, and polyQ domains are boxed, underlined or in grey, respectively. A deletion in clones #1 and #2 (E170_Q176del; *M. minutoides Sry* haplotype *a*, Table 1) resulted in the loss of one polyglutamine block.

```
                   10        20        30        40        50        60        70        80        90       100
Consensus  MEGHVKRPMNAFMVWSCGERQKLAQQNPSMQNTEISKLLGCRWKSLTEGEKRPFFQEAQRLKTLHREKYPNYKYQPHRRAKVPQRSGALQTAVTSTKLYN
Clone#1    ....................................................................................................
Clone#2    ....................................................................................................
Clone#3    ....................................................................................................
Clone#4    ....................................................................................................
Clone#5    ....................................................................................................
Clone#6    ....................................................................................................
Clone#7    ....................................................................................................
Clone#8    ....................................................................................................

                  110       120       130       140       150       160       170       180       190       200
Consensus  LLQWDRNPHTITYRQDWTRAAHLSSKNQQSFYCQPVDIPTGNPLQQQQQQQQQHQFHHHQQQQQHQFHQQQQLKHQHQHQFYQQQQQQHQFHQQQQQQQQ
Clone#1    ....................................................................................................
Clone#2    .......................................................................................-............
Clone#3    ...................................................................------------------------.....
Clone#4    ....................................................................................................
Clone#5    .......................................................................................-............
Clone#6    ...................................................................R................................
Clone#7    ....................................................................................................
Clone#8    ..................................................

                  210       220       230       240       250       260
Consensus  QQQQQQFHQQQQQQQFHQQQQQKQQQQQFHHQHQQQHKQQQHQFHHQQQQKQRQQHQFHQQ
Clone#1    .............................................................
Clone#2    .............................................................
Clone#3    .......................................................R.....
Clone#4    .............................................................
Clone#5    .............................................................
Clone#6    .............................................................
Clone#7    .............................................................
Clone#8
```

Supplementary Figure S4. Alignment of the deduced SRY peptide sequences in *M. mattheyi*. The consensus sequence is displayed above the alignment. Residues identical to the consensus are indicated by dots, while missing residues are depicted by hyphens. The HMG, Bridge, and polyQ domains are boxed, underlined or in grey, respectively. A deletion in clone #3 (Q172_Q195del; *M. mattheyi Sry* haplotype *c*, Table 1) resulted in the loss of two polyglutamine block, while the polyQ tract is almost completely lost in clone #8 (*M. mattheyi Sry* haplotype *g*, Table 1).

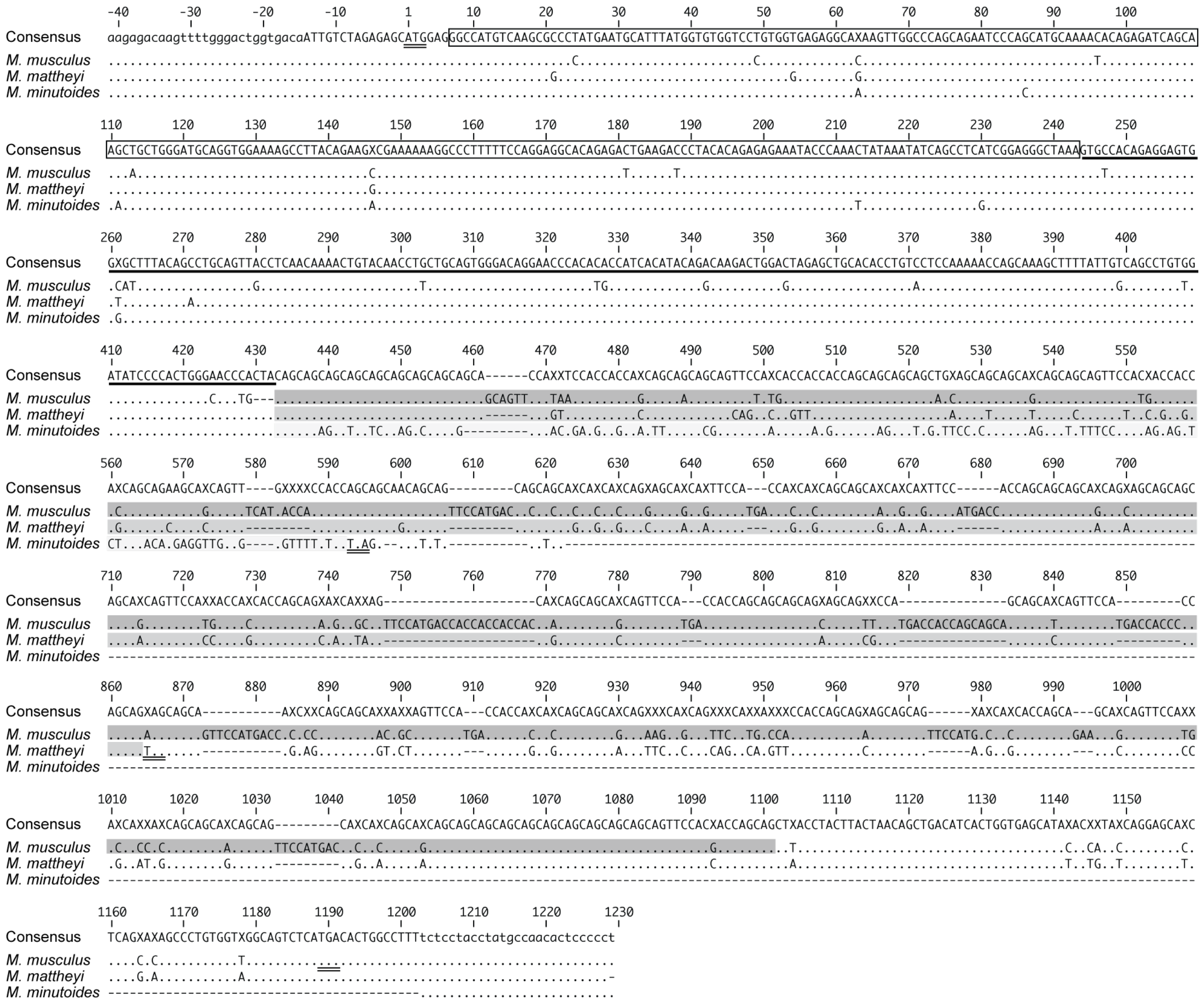

Supplementary Figure S5. Nucleotide sequence comparison of *Sry* sequences in *M. minutoides*, *M. mattheyi*, and *M. musculus*. Reference *Sry* sequences in *M. minutoides* (clone #7, Supplementary Fig S1; see also Table 1) and *M. mattheyi* (clone #4, Supplementary Fig S2) are used here. *M. musculus Sry* sequence is retrieved from mm10 reference genome. The consensus is displayed above the alignment. Sequences identical to the consensus are indicated by dots, while missing nucleotides are depicted by hyphens. Binding sites of PCR primers are in lower case. Start and stop codons are double-underlined. Sequences encoding the HMG, Bridge, and polyQ domains are boxed, underlined or in grey, respectively.

```
                      10        20        30        40        50        60        70        80        90       100
                       |         |         |         |         |         |         |         |         |         |
Consensus     MEGHVKRPMNAFMVWSCGERQKLAQQNPSMQNTEISKLLGCRWKSLTEXEKRPFFQEAQRLKTLHREKYPNYKYQPHRRAKVPQRSGALQPAVTSTKLYN
M. musculus   ................R...H................Q..........A.............I...................S....I.....A......
M. mattheyi   ................................................G.........................................T.........
M. minutoides ............................T...................D...........................R.......................

                     110       120       130       140       150       160       170       180       190       200
                       |         |         |         |         |         |         |         |         |         |
Consensus     LLQWDRNPHTITYRQDWTRAAHLSSKNQQSFYCQPVDIPTGNPLQQQQQQQQQXQFHXHXQQQQ--XXXHQQQQLKXQXQXQFXXXQQQQHQFHXQXQQQ
M. musculus   .........A.......S.....Y........W........-H..........Q...N.H....QFYDH.....QQQ.Q.Q..HDHH..KQ...DHH...
M. mattheyi   .....................................................H...H.Q....--HQF.......H.H.H..YQQ........Q.Q...
M. minutoides ..............................................RLL.--------------------D.R...----------..L..E.QH.R..E

                     210       220       230       240       250       260       270       280       290       300
                       |         |         |         |         |         |         |         |         |         |
Consensus     Q------XXXQQQQFHXXXQQQQ---XHQQQQQXQQQQQFHXXHQQX-------HXQQQXQFHXXQQQX------QRQQHQFHXX---------------
M. musculus   .QFHDHHHHH.E....DHH....QFHD......Q.......DH...KQQFHDHH.H...Q...DH...QQQFHDH.Q......DHPQQKQQFHDHPQQQQ
M. mattheyi   .------QQQ......QQQ....---F......K.......HQ...Q-------.K...H...HQ...K------........QQ
M. minutoides L------VSP..EL.PHS--S----------S.------------------------------------------T.GWWV.S

                     310       320       330       340       350       360       370       380       390
                       |         |         |         |         |         |         |         |         |
Consensus     -----------------------------------------------------------------------------------------------
M. musculus   QFHDHHHQQQQKQQFHDHHQQKQQFHDHHQQKQQFHDHHQQQQQFHDHHQQQQQQQQQQQQFHDQQLTYLLTADITGEHTPYQEHLSTALWLAVS
M. mattheyi
M. minutoides
```

Supplementary Figure S6. Comparison of deduced SRY peptide sequences in *M. musculus*, *M. mattheyi*, and *M. minutoides*. Reference SRY sequences in *M. minutoides* (clone #7, Supplementary Fig S1; see also Table 1) and *M. mattheyi* (clone #4, Supplementary Fig S2) are used here. *M. musculus* SRY sequence is retrieved from mm10 reference genome. The consensus sequence is displayed above the alignment. Residues identical to the consensus are indicated by dots, while missing residues are depicted by hyphens. The HMG, Bridge, and polyQ domains are boxed, underlined or in grey, respectively. * indicates an amino acid substitution (R17C) in *M. mattheyi* and *M. minutoides*, compared with *M. musculus*.

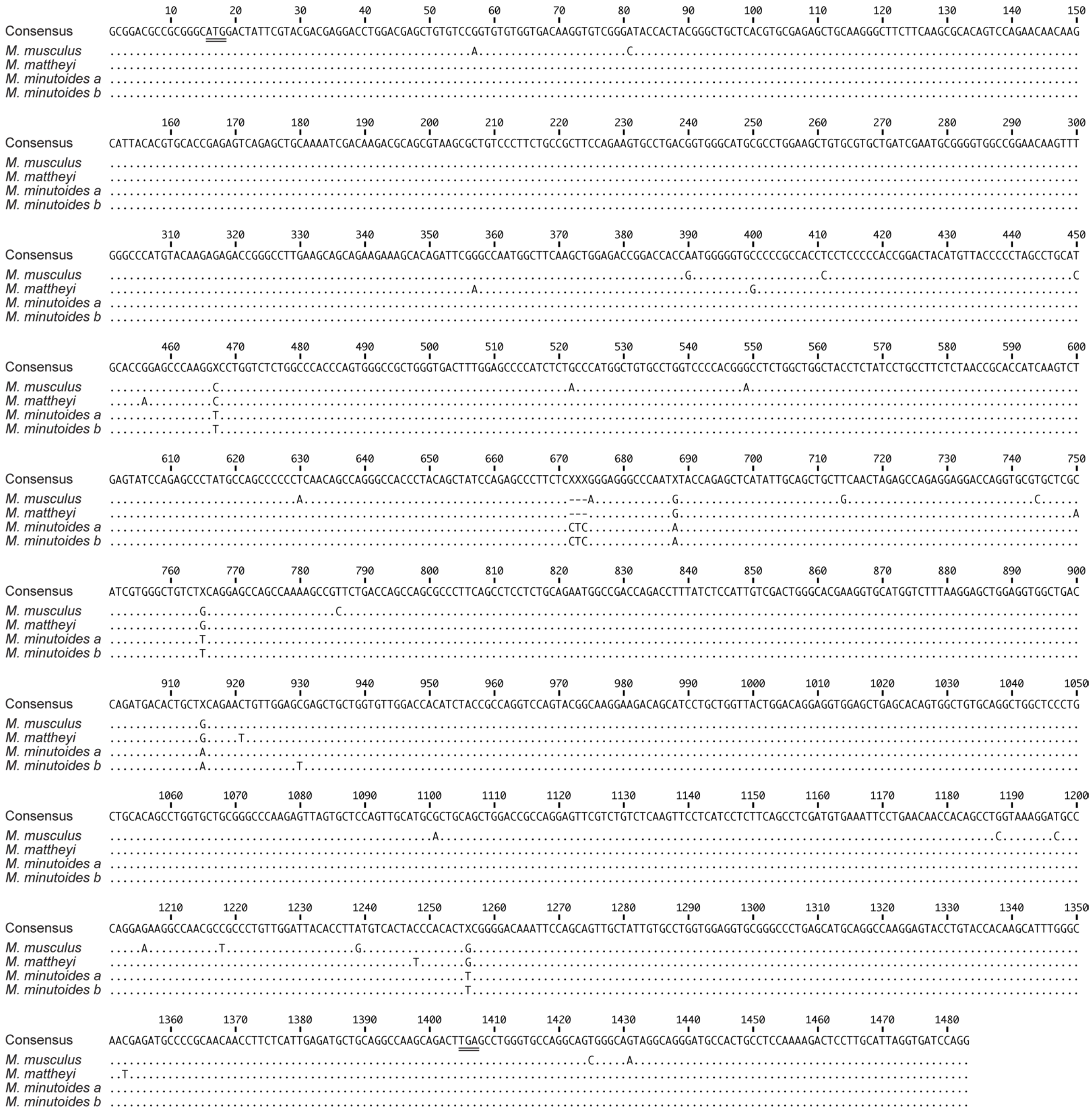

Supplementary Figure S7. Nucleotide sequence alignment of *Sf1* coding sequences in *M. musculus*, *M. mattheyi*, and *M. minutoides*. *M. mattheyi* and *M. minutoides Sf1* coding sequences are assembled from sequencing results of individual *Sf1* exons 2–7. The *M. mattheyi* individual analysed appeared to carry two identical *Sf1* alleles, whereas two different *Sf1* alleles (*a*, *b*) were identified in the *M. minutoides* individual. *M. musculus Sf1* sequence is retrieved from mm10 reference genome. The consensus sequence is displayed above the alignment. Sequences identical to the consensus are indicated by dots, while missing nucleotides are depicted by hyphens. Start and stop codons are double-underlined.

```
                       10        20        30        40        50        60        70        80        90       100
Consensus      MDYSYDEDLDELCPVCGDKVSGYHYGLLTCESCKGFFKRTVQNNKHYTCTESQSCKIDKTQRKRCPFCRFQKCLTVGMRLEAVRADRMRGGRNKFGPMYK
M. musculus    ....................................................................................................
M. mattheyi    ....................................................................................................
M. minutoides  ....................................................................................................

                      110       120       130       140       150       160       170       180       190       200
Consensus      RDRALKQQKKAQIRANGFKLETGPPMGVPPPPPPPPDYMLPPSLHAPEPKALVSGPPSGPLGDFGAPSLPMAVPGPHGPLAGYLYPAFSNRTIKSEYPEP
M. musculus    ....................................................................................................
M. mattheyi    .............................A......................................................................
M. minutoides  ......................................................................................................V.................................................
```

```
                      210       220       230       240       250       260       270       280       290       300
Consensus      YASPPQQPGPPYSYPEPFS-GGPNVPELILQLLQLEPEEDQVRARIVGCLQEPAKSRSDQPAPFSLLCRMADQTFISIVDWARRCMVFKELEVADQMTLL
M. musculus    ...................-................................................................................
M. mattheyi    ...................-................................................................................
M. minutoides  ...................S....I...........................................................................

                      310       320       330       340       350       360       370       380       390       400
Consensus      QNCWSELLVLDHIYRQVQYGKEDSILLVTGQEVELSTVAVQAGSLLHSLVLRAQELVLQLHALQLDRQEFVCLKFLILFSLDVKFLNNHSLVKDAQEKAN
M. musculus    ....................................................................................................
M. mattheyi    ....................................................................................................
M. minutoides  ....................................................................................................

                      410       420       430       440       450       460
Consensus      AALLDYTLCHYPHCGDKFQQLLLCLVEVRALSMQAKEYLYHKHLGNEMPRNNLLIEMLQAKQT
M. musculus    ...............................................................
M. mattheyi    ...............................................................
M. minutoides  .............F.................................................
```

Supplementary Figure S8. Deduced peptide sequence alignment of SF1 in *M. musculus*, *M. mattheyi*, and *M. minutoides*. Note the two *M. minutoides Sf1* alleles (S7 Fig) encode the same protein. *M. musculus* SF1 sequence is retrieved from mm10 reference genome. The consensus sequence is displayed above the alignment. Residues identical to the consensus are indicated by dots, while missing residues are depicted by hyphens.

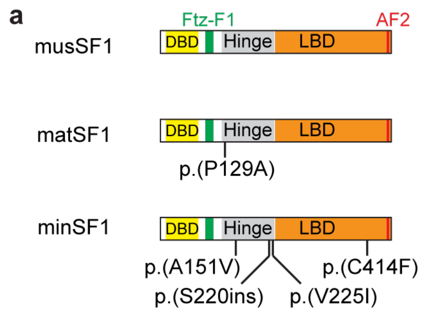

c

Two-way ANOVA results (panel b)

Factor	% of total variation	F	*P*
SRY	97.54	690.0	<0.0001
SF1	0.47	4.575	0.0925
Interaction	0.20	0.648	0.7572

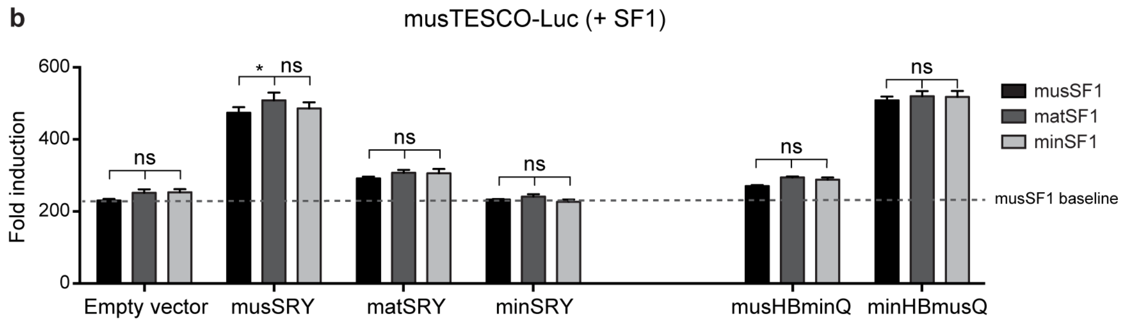

Supplementary Figure S9. SF1 proteins from *M. musculus*, *M. mattheyi*, and *M. minutoides* show similar activities in musTESCO-Luc reporter assays. (a) Schematic of SF1 proteins from three *Mus* species: *M. musculus* (musSF1), *M. mattheyi* (matSF1), and *M. minutoides* (minSF1). DBD, DNA binding domain; LBD, ligand-binding domain. Non-synonymous sequence changes compared with musSF1 are indicated in matSF1 and minSF1. (b) Synergistic activation of musTESCO-Luc by various combinations of SRY (including two chimeric mutants) and SF1 from *M. musculus*, *M. mattheyi*, and *M. minutoides* were tested using *in vitro* reporter assays. The luciferase activity of musTESCO-Luc co-transfected with the empty vector in the absence of SF1 was set to 1. For simplicity, only the +SF1 data are presented here as mean ± s.e.m (n = 3), as the –SF1 data essentially showed unchanged base level activities of musTESCO-Luc reporter. Dashed line indicates the level of empty vector + musSF1. (*) $P < 0.05$, Tukey's multiple comparisons test. ns, not significant. (c) Summary of two-way repeated measures ANOVA results of (b).

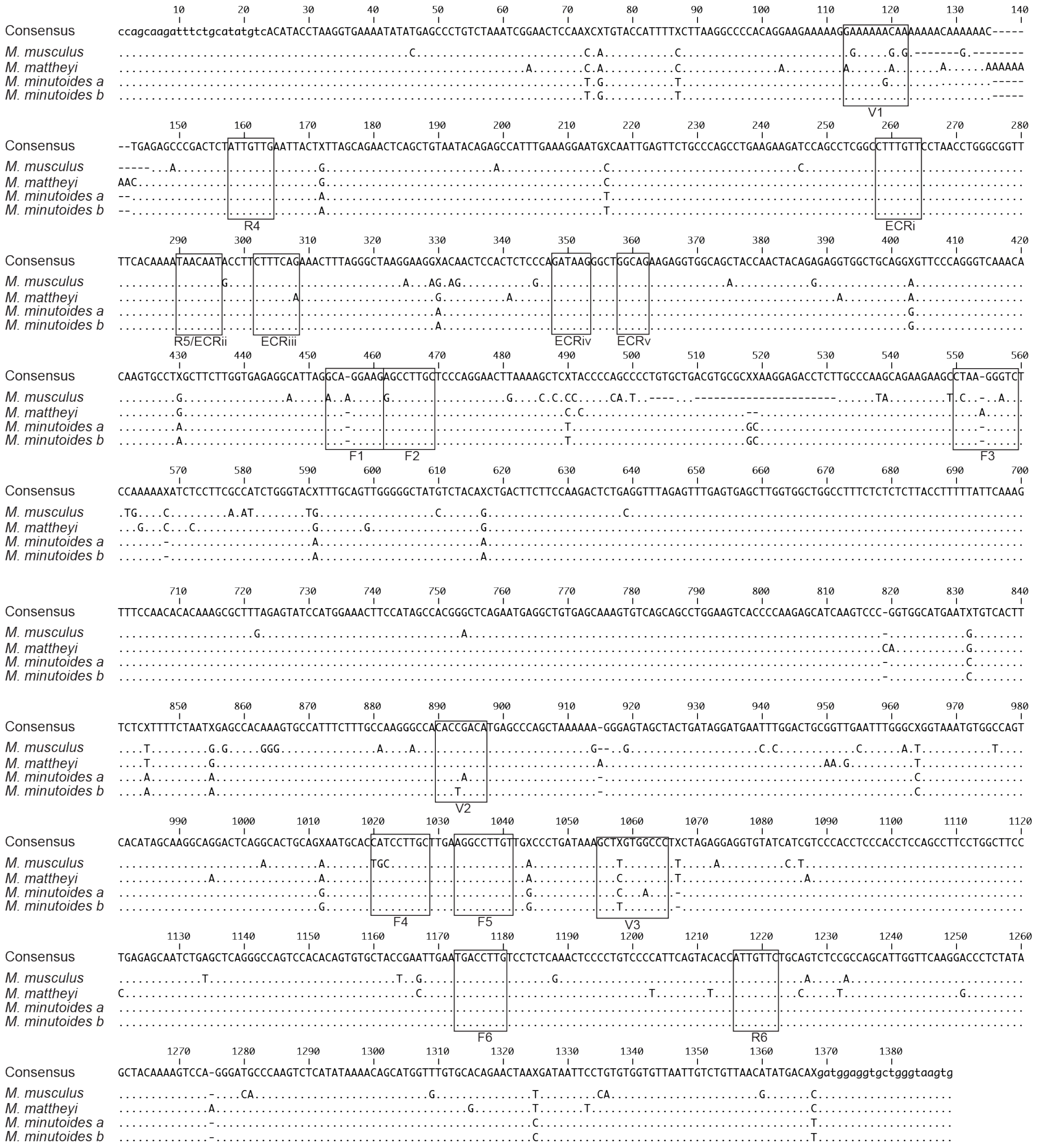

Supplementary Figure S10. Nucleotide sequence alignment of TESCO sequences in *M. musculus*, *M. mattheyi*, and *M. minutoides*. The *M. mattheyi* individual analysed appeared to carry two identical copies of TESCO, whereas two TESCO haplotypes were identified in *M. minutoides*. The binding sites of SRY and SF1 identified in *M. musculus* (R4-5 and F1-6, respectively), the evolutionarily conserved sites (ECRi-v), and the sequence variations between the two haplotypes of minTESCO (V1-3) are highlighted in boxes. SRY binding sites and most of the ECR sites remain

unchanged in mat/minTESCO, whereas several SF1 binding sites showed sequence variations in *M. mattheyi* and *M. minutoides*. The consensus sequence is displayed above the alignment. Identical residues are indicated by dots, while missing residues are depicted by hyphens. Binding sites of PCR primers are in lower case.

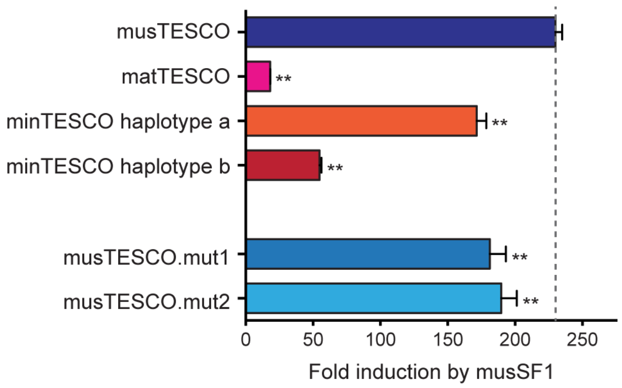

Supplementary Figure S11. TESCO enhancer in *M. minutoides* and *M. mattheyi* showed reduced response to musSF1. Mutations of SF1 binding sites F1-4 in musTESCO to the corresponding sequence in matTESCO (musTESCO.mut1-Luc) or minTESCO (musTESCO.mut2-Luc) caused mildly reduced response to musSF1. Data are presented as fold induction in the presence versus the absence of musSF1. Error bars: s.e.m. ($n = 3$). Dashed line indicates the level of musTESCO. (**) $P < 0.01$ vs. musTESCO, one-way repeated measures ANOVA with Dunnett's multiple comparisons test.

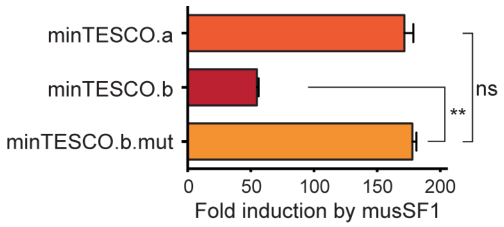

Supplementary Figure S12. Sequence variations at the V3 site between two haplotypes of *M. minutoides* TESCO results in reduced response to musSF1. minTESCO.b.mut with V3 site mutated to the corresponding sequence in minTESCO.a showed fully restored response to musSF1. Data are presented as TESCO luciferase activity normalized to co-transfected CMV-renilla luciferase activity. Error bars: s.e.m. (n = 3). (**) $P < 0.01$, one-way repeated measures ANOVA with Dunnett's multiple comparisons test. ns, not significant.

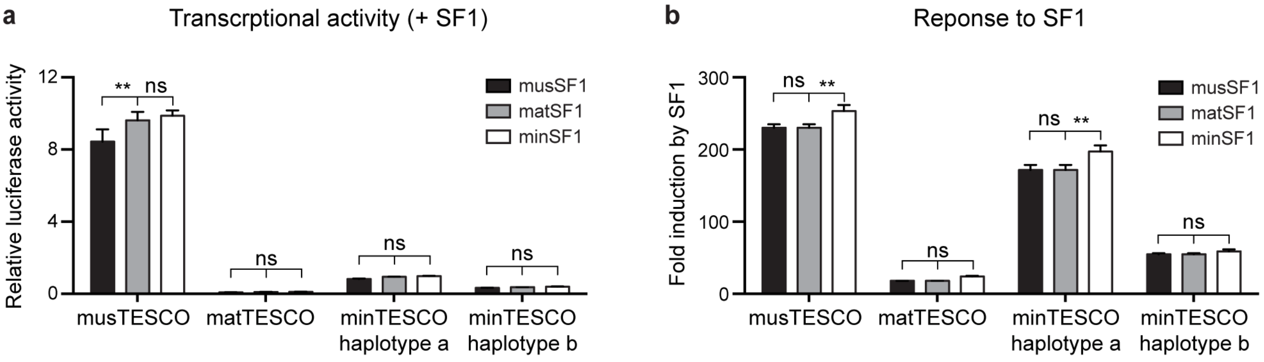

c

Two-way ANOVA results

Factor	Panel a % of total variation	Panel a F	Panel a *P*	Panel b % of total variation	Panel b F	Panel b *P*
TESCO	98.48	369.8	<0.0001	98.5	931.6	<0.0001
SF1	0.2217	15.13	0.0136	0.603	53.83	0.0013
Interaction	0.4397	9.454	0.0006	0.2651	4.342	0.0147

Supplementary Figure S13. SF1 proteins from *M. musculus*, *M. mattheyi*, and *M. minutoides* show similar activities in min/matTESCO reporter assays. (a,b) Activation of TESCO by SF1 from three Mus species were tested using *in vitro* reporter assays. Different TESCO reporter constructs contribute to > 98% of total variation (c). Compared with matSF1 and musSF1, minSF1 showed mildly higher activation of both musTESCO and minTESCO.a, but not minTESCO.b, suggesting that *Sf1* is unlikely to have co-evolved with TESCO in *M. minutoides* (see also Supplementary Fig. S14). The luciferase activity of each TESCO-Luc co-transfected with the empty vector in the absence of SF1 was set to 1. Error bars: s.e.m (n=3). (**) $P < 0.01$, Tukey's multiple comparisons test. ns, not significant. (c) Summary of two-way repeated measures ANOVA results of (a,b).

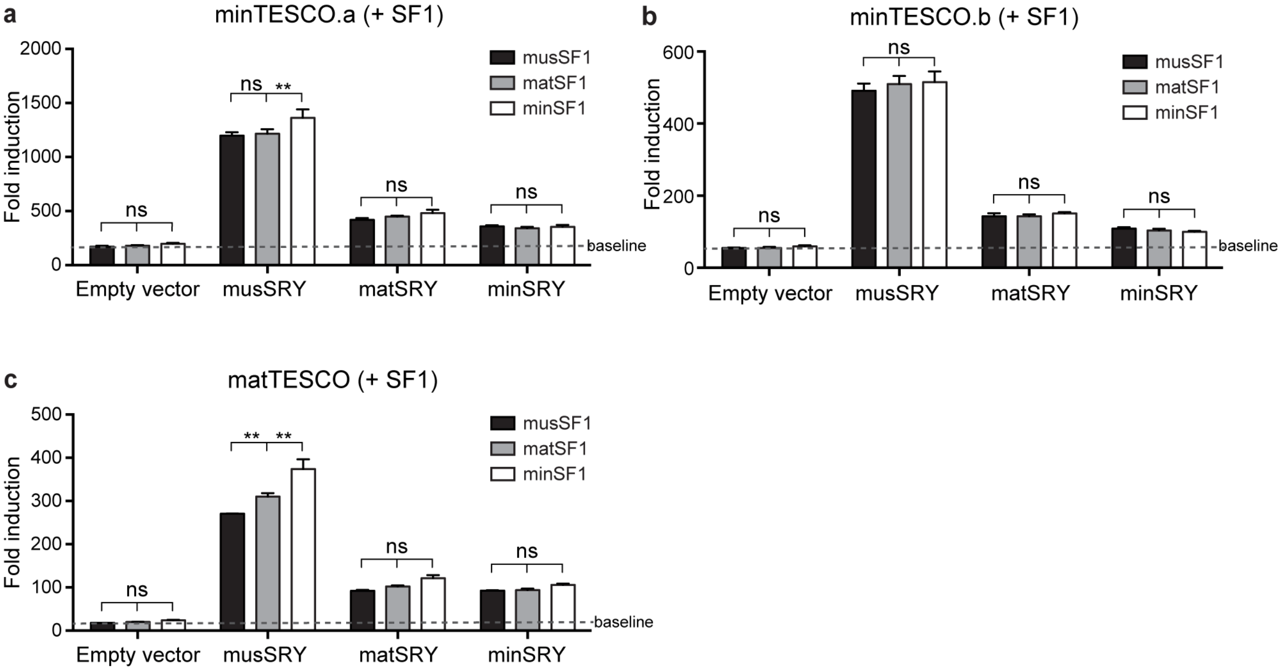

d Two-way ANOVA results

Factor	Panel a (minTESCO.a)			Panel b (minTESCO.b)			Panel c (matTESCO)		
	% of total variation	F	*P*	% of total variation	F	*P*	% of total variation	F	*P*
SRY	98.05	1208	<0.0001	98.87	505.2	<0.0001	95.26	1440	<0.0001
SF1	0.42	2.277	0.2187	0.02	0.479	0.6512	1.93	13.41	0.0168
Interaction	0.47	3.575	0.0287	0.08	0.987	0.4756	1.97	12.70	0.0001

Supplementary Figure S14. SF1 proteins from *M. musculus*, *M. mattheyi*, and *M. minutoides* show similar activities in synergizing with different SRY in activating various TESCO reporters. Synergistic activation of different TESCO luciferase reporters by various combinations of SRY and SF1 from *M. musculus*, *M. mattheyi*, and *M. minutoides* were tested using *in vitro* reporter assays (a-c). With all three TESCO reporters, different SRY constructs contribute to > 95% of total variation (d). Compared with matSF1 and musSF1, minSF1 showed mildly higher activation of minTESCO.a-Luc (a) and matTESCO-Luc (c) in the presence of musSRY. However, minSF1 did not outperform musSF1 or matSF1 in activating its cognate minTESCO.a/b-Luc reporters in the presence of cognate minSRY, suggesting that *Sf1* is unlikely to have co-evolved with *Sry* or TESCO in *M. minutoides*. The luciferase activity of each TESCO-Luc co-transfected with the empty vector in the absence of SF1 was set to 1. For simplicity, only the +SF1 data are

presented here as mean ± s.e.m (n=3). Dashed lines indicate the levels of empty vector + musSF1. (**) $P < 0.01$, Tukey's multiple comparisons test. ns, not significant. (d) Summary of two-way repeated measures ANOVA results of (a-c).

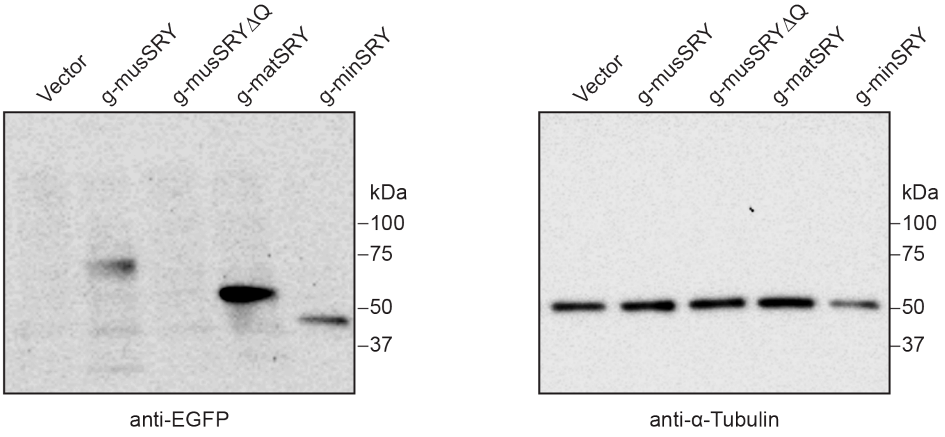

Supplementary Figure S15. Full-length Western blots in Fig. 2b. Predicted molecular weight: g-musSRY, 77.1 kDa; g-musSRY ΔQ, 47.9 kDa; g-minSRY, 50.7 kDa; g-matSRY, 59.9 kDa; α-Tubulin, 50 kDa.

Supplementary Table S1. PCR primers.

Primer	Sequence	Note
Sry.Fw	AGATCTAAGAGACAAGTTTTGGGACTGGTGACA	*Bgl*II site underlined
Sry.Rv	CTCGAGAGGGGGAGTGTTGGCATAGGTAGGAGA	*Xho*I site underlined
TESCO.Fw	CCAGCAAGATTTCTGCATATGTC	
TESCO.Rv	CACTTACCCAGCACCTCCATC	
Sf1exon2_3.Fw	agGCGGACGCCGCGGGCATGGACTATT	Intronic sequence in small letters
Sf1exon2_3.Rv	acCTTCCAGGCGCATGCCCACCGTCA	
Sf1exon4.Fw	agCTGTGCGTGCTGATCGAATG	Intronic sequence in small letters
Sf1exon4.Rv	acCTCCAGCTCCTTAAAGACCATGCA	
Sf1exon5.Fw	agGTGGCTGACCAGATGACACTGCT	Intronic sequence in small letters
Sf1exon5.Rv	acCTCCTGTCCAGTAACCAGCAGGAT	
Sf1exon6.Fw	agGTGGAGCTGAGCACAGTGGCTGT	Intronic sequence in small letters
Sf1exon6.Rv	acCGAGGCTGAAGAGGATGAGGAACTT	
Sf1exon7.Fw	gcagATGTGAAATTCCTGAACAACCACA	Intronic sequence in small letters
Sf1exon7.Rv	CCTGGATCACCTAATGCAAGGAGTCTT	
